# Iguratimod as an alternative therapy for systemic sclerosis and prevention of the occurrence of ischemic digital ulcer

**DOI:** 10.3389/fmed.2023.1113408

**Published:** 2023-05-05

**Authors:** Qingran Yan, Bei Liu, Jieying Wang, Hanlin Yin, Qianqian Li, Liangjing Lu

**Affiliations:** ^1^Department of Rheumatology, Renji Hospital, Shanghai Jiaotong University School of Medicine, Shanghai, China; ^2^Clinical Center for Investigation, Renji Hospital, Shanghai Jiaotong University School of Medicine, Shanghai, China

**Keywords:** systemic sclerosis, iguratimod, digital ulcer, alternative therapy, prevention

## Abstract

**Objectives:**

This study aimed to assess the effectiveness of iguratimod (IGU) as an alternative treatment for systemic sclerosis (SSc), especially in the prevention of ischemic digital ulcers (DUs).

**Methods:**

We constructed two cohorts from the Renji SSc registry. In the first cohort, SSc patients receiving IGU were observed prospectively with effectiveness and safety. In the second cohort, we picked up all the DU patients with at least a 3-month follow-up to investigate the prevention of IGU on ischemic DU.

**Results:**

From 2017 to 2021, 182 SSc patients were enrolled in our SSc registry. A total of 23 patients received IGU. With a median follow-up of 61 weeks (IQR: 15–82 weeks), the drug persistence was 13/23. In total, 91.3% of the patients (21/23) became free of deterioration in the last visit with IGU. Of note, 10 patients withdrew from the study due to the following reasons: two patients withdrew due to deterioration, three due to incompliance, and five due to mild-to-moderate side effects. All the patients with side effects recovered fully after stopping IGU. Of note, 11 patients had ischemic DU, and 8 out of 11 (72.7%) patients had no new occurrence of DU during the follow-up. In the second cohort of 31 DU patients receiving a combination of vasoactive agents with a median follow-up of 47 weeks (IQR, 16–107 weeks), IGU treatment was protective of new DU occurrence (adjusted risk ratio = 0.25; 95% CI, 0.05–0.94; adjusted odds ratio = 0.07; and 95% CI, 0.01–0.49).

**Conclusion:**

Our study for the first time describes the potential of IGU possibly as an alternative treatment for SSc. To our surprise, this study provides a hint that IGU treatment can be used for the prevention of the occurrence of ischemic DU and merits further investigation.

## Introduction

Systemic sclerosis (SSc) is an autoimmune disease that affects a variety of tissues and organs and is characterized by immune disorders, microvascular damage, and fibrosis (1, 2). The pathogenesis is complicated and unclear. Immunosuppression is a major strategy to treat SSc, especially for inflammatory and fibrotic symptoms, such as skin thickening (3, 4), interstitial lung disease (ILD) (5–7), heart involvement, and arthritis. Although a massive advance in treatment has been achieved for SSc patients, given the high heterogenicity of both clinical manifestation and disease progression, unmet therapeutic requests are still the central issue of SSc. Not only is SSc one of the highest lethality autoimmune diseases, but it can also increase the burden of living due to non-fatal complications such as digital ulcers (8, 9).

Iguratimod (IGU) is a novel anti-rheumatic medicine that has been licensed in East Asia as a disease-modifying anti-rheumatic drug (DMARD) for the treatment of rheumatoid arthritis (RA) (10). In the phase III clinical trial performed in Japan, IGU showed superiority over placebo and non-inferiority to salazosulfapyridine (SASP) (11). Our team completed the phase III clinical trial in China and found that IGU was non-inferior to methotrexate (MTX), with fewer and milder side effects (12). A post-marketing surveillance study involving more than 2,000 patients for 52 weeks provided real-world evidence that IGU was safe and effective in RA patients (13). In addition, IGU showed add-on efficacy in RA patients with inadequate response to methotrexate (14), methotrexate–cyclosporin A–hydroxychloroquine–prednisone (15), or biological DMARDs (16, 17).

IGU is typically thought of as an anti-inflammatory and immunoregulatory drug that can reduce the production of inflammatory cytokines, such as interleukin (IL)-1, IL-6, IL-8, and tumor necrosis factor (TNF), regulate the subpopulation of B cells, decrease the production of immunoglobulin, and stop the proliferation of pro-inflammatory T cells (18). On top of anti-inflammation, IGU has shown potential efficacy on fibrotic complications with autoimmune diseases. We and other researchers previously showed that IGU ameliorated bleomycin (BLM)-induced pulmonary and cutaneous fibrosis in animal experiments (19, 20). In a preliminary observation for RA-ILD, IGU showed a possible benefit to lung function (21). This study aimed to describe the effectiveness and safety of IGU treatment in our SSc registry patients.

## Methods

### Patients and study design

All patients were selected from a prospective, observational SSc registry of Renji Hospital, established in 2017. Patients with other co-existing autoimmune diseases, such as systemic lupus erythematosus (SLE), Sjögren's syndrome (SS), or rheumatoid arthritis (RA), were excluded. ILD patients in this registry were all confirmed by high-resolution computed tomography (HRCT). Pulmonary hypertension (PH) was diagnosed by right heart catheter or echocardiography (peak tricuspid regurgitation velocity of >3.4 m/s). This study was approved by the Ethics Committee of Renji Hospital, Shanghai, China.

Of the 182 patients in the SSc registry of Renji Hospital, we constructed two cohorts. Participants gave written informed consent. Patients included in the two cohorts of this study were required to fulfill the 2013 ACR/EUALR or 1980 ACR classification criteria (22). The first cohort includes all those who received IGU from March 2017 to March 2022. The baseline information at the start date of IGU, reasons for IGU use, and follow-up information were analyzed. Disease worsening would be recorded with the presence of at least one of the following: ≥25% increase in mRSS and an increase in mRSS of >5 points; a new renal crisis; a new ILD on HRCT; an absolute decline in FVC of ≥ 10% predicted; a new onset of the left ventricular heart failure (LVEF ≤45%); a new onset of pulmonary hypertension (on RHC); a significant gastrointestinal dysmotility requiring parenteral or enteral nutrition; or significant digital ischemia with gangrene or requiring amputation (23–27). The addition of any anti-fibrotic treatment, such as nintedanib during IGU treatment, would also be considered as disease worsening.

The second cohort was created to investigate the preventative effect of IGU on DU occurrence. All patients should have at least a 3-month follow-up record of DU. The ischemic DU was defined as located at or distal to the proximal interphalangeal joint, present in the case of a break in the skin with a loss of epithelialization, according to the physician, and not located over subcutaneous calcifications or over extensor surfaces of joints (28–30). The follow-up started for those who received the IGU treatment, and it started from the inclusion date of the Renji registry if the patients did not receive IGU. The primary endpoint of observation was any new occurrence of ischemic DU during the follow-up.

### Statistical analysis

The baseline clinical characteristics of the study participants were summarized using medians with IQRs for continuous variables and proportions for categorical outcomes. The Mann–Whitney test was used for group comparison of continuous variables. Fisher's exact chi-squared test or the likelihood-ratio test was used for group comparison of categorical outcomes. The alternative hypothesis was accepted at a statistical significance level of *p* < 0.05 on all applied statistical tests. Analyses were conducted using IBM SPSS Statistics 25.0. A multivariate analysis using Poisson regression was used to calculate adjusted relative risk (RR) with 95% CI. Logistic regression was used to calculate the adjusted odds ratio (OR) with 95% CI. Data were analyzed using R language software (Version R 4.2.1).

## Results

### IGU was a plausible alternative treatment for SSc

Of the 182 patients in the Renji SSc registry, 23 had a history of IGU treatment. They were 20 women and three men. The median age of the patients in the study group was 53.0 years (IQR, 37.0–66.0 years). The median duration of SSc was 3.0 years (IQR: 2.0–9.0 years). In total, 11 of these patients had diffuse cutaneous systemic sclerosis (dcSSc), 11 of them had limited cutaneous systemic sclerosis (lcSSc), and one patient had sine SSc. IGU was a secondary treatment in most cases (21/23). The most common justification for initiating IGU treatment was an unsatisfactory control of cutaneous symptoms, synovitis, ILD, or intolerance of previous immunosuppressants (IS). For all these 23 patients, IGU was used with no other IS or biologics. In all patients, the dosage of glucocorticoids was <10 mg daily. Major clinical characteristics are shown in Table 1.

**Table 1:** Demographic information, clinical features, and major outcomes for all patients.

**Pati ent No**.	**Age**	**Sex**	Duration^*^(years)	**Skin type**	**Clinical features #**	**Antibodies**	**Previous IS**	**Reason for drug switch**	**Duration of IGU (weeks)**	**Combined drugs**	**Outcome of manifestations**	**Reason for discontinuing**
1	37	M	3	dcSSc	DU, ILD, PH	Scl-70	MMF	Cutaneous activation	96	Prednisone, sildenafil	mRSS improved§, FVC improved^†^, DU healed (new occurrence), PH stable	Follow-up
2	55	F	3	lcSSc	Synovitis^‡^	CENP B	MTX	Flare ofsynovitis	48	/	Synovitis improved	Follow-up
3	49	F	1	dcSSc	DU, ILD	Scl-70	MMF	Declined FVC	17	Prednisone, nifedipine, sildenafil	FVC decrease ≥ 10%, mRSS progressed, DU healed (no occurrence)	Disease worsening: mRSS and FVC
4	53	F	9	dcSSc	DU, ILD,	Scl-70	CYC	Cutaneous activation	39	Prednisone, sildenafil, bosentan	mRSS improved, DU healed (newoccurrence), FVC decrease 5%~10%	Follow-up
5	68	F	10	dcSSc	DU, PH, ILD	nRNP	CYC	DU infection	174	Prednisone, bosentan	DU healed (no occurrence), PH andFVC stable*	Follow-up
6	31	F	3	lcSSc	DU, ILD	ANA alone	MTX	Declined FVC	37	Prednisone, aspirin, BPS	FVC stable, no occurrence of DU	Incompliance
7	66	F	3	lcSSc	ILD	CENP B	MTX	Worsening of respiratorysymptoms	4	Prednisone, BPS	Symptoms improved, FVC stable	Adverse event: abnormal liverfunction
8	71	F	2	lcSSc	Synovitis	ANA alone	MTX	No remission of synovitis	74	Prednisone, sildenafil, BPS	Synovitis relieved	Follow-up
9	35	F	2	dcSSc	DU, ILD, PH, synovitis	nRNP	CYC	Cutaneousactivation	57	Prednisone, bosentan	mRSS stable, no occurrence of DU, synovitis relieved, FVC stable	Adverse event:anemia
10	52	F	1	lcSSc	None	CENP B	MTX	Cutaneousactivation	69	Prednisone, BPS	mRSS stable	Follow-up
11	71	F	1	sine SSc	PH	CENP B	None	Aggravation of puffy fingers	65	Prednisone, sildenafil, ambrisentan	Puffy fingers improved, PH stable	Follow-up
12	64	F	13	lcSSc	ILD	CENP B	None	Declined FVC	56	Prednisone, sildenafil, nifedipine	FVC improved	Follow-up
13	67	F	7	lcSSc	Synovitis	CENP B	MTX	MTXintolerance	146	Prednisone, nifedipine	Sustained remission	Follow-up
14	53	F	5	lcSSc	DU	nRNP	MTX	Cutaneous activation	8	Prednisone, sildenafil	mRSS stable, DU healed (no occurrence)	Adverse event: abnormal liverfunction
15	33	F	4	lcSSc	Synovitis	CENP B	MTX	Newly-onsetsynovitis	78	Prednisone, nifedipine	Synovitis relieved	Follow-up
16	25	F	5	lcSSc	Synovitis	nRNP	MTX	Flare ofsynovitis	78	Prednisone, BPS	Synovitis improved	Adverse event:GI intolerance
17	56	M	2	dcSSc	DU, ILD	Scl-70	CYC	Declined FVC, cutaneousactivation	10	Prednisone, sildenafil	FVC decrease≥ 10%, mRSS improved, DU healed (nooccurrence)	Disease worsening: FVC
18	37	F	13	dcSSc	DU, ILD	ANA alone	MMF	DU infection	8	Prednisone, nintedanib, sildenafil	DU healed (no occurrence), FVC improved	Incompliance
19	32	F	12	dcSSc	DU, synovitis	nRNP	CYC	Synovitis flare	145	Prednisone, aspirin, tadalafil	Synovitis improved, DU healed (new occurrence)	Follow-up
20	61	F	3	dcSSc	ILD, PH, synovitis	nRNP	MMF	Cutaneousactivation	107	Prednisone, aspirin, BPS	mRSS improved, synovitis relieved, FVC improved, PH stable	Follow-up
21	52	F	1	lcSSc	ILD, synovitis	Scl-70	CYC	No remissionof synovitis	9	Nintedanib	Synovitis relieved, FVC improved	Adverse event:urticaria
22	68	F	11	dcSSc	ILD, DU, synovitis	Scl-70	CYC → AZA	Declined FVC, no remission of synovitis	77	Sildenafil, aspirin, ambrisentan	FVC improved, synovitis improved, mRSS improved, DU healed (no occurrence)	Incompliance
23	44	M	1	dcSSc	Myositis	PM-Scl	MTX → Tocilizumab	ensuing of 2 courses oftocilizumab	43	Prednisone	myositis relieved, mRSS improved	Follow-up

* SSc disease duration was determined as the time from the onset of the first non-Raynaud's phenomenon (RP) manifestation.

^#^Clinical features refer to symptoms other than RP, including ILD, DU, PH, synovitis, and myositis, as RP is present in every patient.

^§^mRSS improvement, for dcSSc, mRSS decreased≥5 and by≥25% relative to baseline; for lcSSc, mRSS decreased≥4 (31).

† Lung function improvement required an absolute increase in predicted FVC >5%. ^*^The change of recent predicted FVC from baseline between 5% improved and 5% decreased.

‡ Either tender or swollen joints were considered synovitis (25). Improvement required a reduction of ≥20% counts in tender and swollen joints. lcSSc, limited cutaneous systemic sclerosis; dcSSc, diffuse cutaneous systemic sclerosis; DU, digital ulcers; PH, pulmonary hypertension; ILD, interstitial lung disease; ANA, antinuclear antibody; anti-Scl-70, anti-topoisomerase I; RP, Raynaud phenomenon; BPS, beraprost sodium; ERA, endothelin receptor antagonist.

During the follow-up with a median of 61 weeks (IQR: 15–82 weeks), the IGU persistence was 56.5% (13/23). Overall, 91.3% of the patients (21/23) had no deterioration until the last visit with IGU. In total, 10 patients had synovitis, and all of them had improvement or remission. Of 13 patients with HRCT-confirmed ILD, an improvement of >5% in predicted FVC was observed in six patients, FVC change between 5% improved and 5% decreased was observed in four patients, FVC decreased between 5% and 10% was observed in one patient, and two patients had an absolute decrease in FVC of ≥ 10%. In total, 11 patients had ischemic Dus; of which, nine of them had current ischemic DUs at the start date of IGU. All nine current DU patients healed, and unexpectedly, 8 out of 11 (72.7%) patients had no new DU occurrence during the follow-up. No escalation of risk stratification for PH was observed.

A total of 10 patients withdrew from the study. Of which, two patients withdrew due to deterioration: one worsening case had declined FVC, and another had both worsening FVC and mRSS. Both discontinued IGU. In total, 3 out of 10 patients discontinued IGU because of incompliance, two of them stopped IGU because of satisfactory disease control (pt6 and pt22), and one was unwilling to use IGU (pt18). Regarding safety issues, five out of 10 patients had adverse events during IGU administration. We stopped IGU for all of them due to the most cautious safety concerns. Two patients had elevated alanine transaminase less than three times the upper normal limit, one had gastrointestinal (GI) intolerant, one had mild anemia, and one had urticaria. The anemia, GI intolerance, and urticaria were all late onsets. All these patients recovered fully after discontinuing IGU, with or without symptomatic treatment. There was no infection case.

### IGU in preventing ischemic DU occurrence

To confirm the effect of ischemic DU in the IGU cohort, we constructed the second cohort of 31 patients with current or historical ischemic DU from the Renji SSc registry. The clinical and demographic characteristics of patients are shown in Table 2. Every patient had at least a history of the usage of vasodilators during follow-up. With a median follow-up of 47 weeks (IQR, 16–107 weeks), three (27.3%) patients in the IGU arm had new DU occurrence, while 15 (75%) patients without IGU had DU occurrence. There were no statistical differences in demographics. In dichotomous analysis, only new DU occurrences were statistically different between the IGU group and non-IGU group (P=0.021). Most known risk factors for DU occurrence (sex, skin subtype, disease onset age, ESR, autoantibodies, ILD, smoke, and PH) were not significantly different between the IGU group and the non-IGU group, as shown in Table 2.

**Table 2:** Baseline characteristics of the study participants with digital ulcers.

**Characteristics**	**With IGU (N=11)**	**Without IGU (N=20)**	***p*-value**
**Age of Onset (years), median (IQR)**	36.0 (31.0–53.0)	43.0 (34.0–54.0)	0.457
**Sex (%)**			0.676
Male Female	2 (18.2) 9 (81.8)	6 (30.0) 14 (70.0)	
**Time since RP onset (years), median**			
**(IQR)**	9.0 (3.0–12.0)	7.5 (3.0–14.8)	0.740
**Time since first non-RP**			
**manifestation (years), median (IQR)**	9.0 (2.0–11.0)	5.5 (2.3–12.8)	0.634
**SSc subtype (%)**			0.133
Diffuse Limited Sine scleroderma	9 (81.8) 2 (18.2) 0	10(50.0) 8(40.0) 2(10.0)	
**ESR(mm/h), median (IQR)**	29.0 (15.5–53.8)	27.0 (13.5–47)	0.880
**Autoantibodies(%)**			0.332
Anti-Scl70 antibody positive ACA positive Anti-U1RNP positive Anti-Th/To positive	6 (60.0) 0.332 1 (10.0) 3 (30.0) 0	12 (75.0) 2 (12.5) 1 (6.3) 1 (6.3)	
**Smoke(>25 pack-years)(%)**	0	4 (20.0)	0.269
**Previous DU(s)**	4 (36.4)	8 (40.0)	1.0
**Vasodilator use (at least one agent) (%)**	11 (100)	20 (100)	
PDE5i ERA CCB Beraprost	6 (54.5) 5 (45.5) 4 (36.4) 5 (45.5)	14 (70.0) 5 (25.0) 6 (30.0) 11 (55.0)	0.452 0.423 1 0.716
**Pulmonary hypertension(%)**	3 (27.3)	6 (30.0)	1
**ILD(%)**	9 (81.8)	13 (65.0)	0.429
**DU new occurrence during follow-up(%)**			**0.021**
never new occurrence	8 (72.7) 3 (27.3)	5 (25.0) 15 (75.0)	

DU, digital ulcer; SD, standard deviation; IQR, interquartile range; ESR, erythrocyte sedimentation rate; RP, Raynaud's phenomenon; ANA, antinuclear antibodies; ACA, anticentromere antibody; anti-U1RNP, antiribonucleoprotein antibodies; IGU, iguratimod; SSc, systemic sclerosis; anti-Scl70, anti-topoisomerase I; PAH, pulmonary arterial hypertension; PDE5i, phosphodiesterase type-5 inhibitors; ERA, endothelin receptor antagonist; CCB, calcium channel blocker.

**Figure 1:**
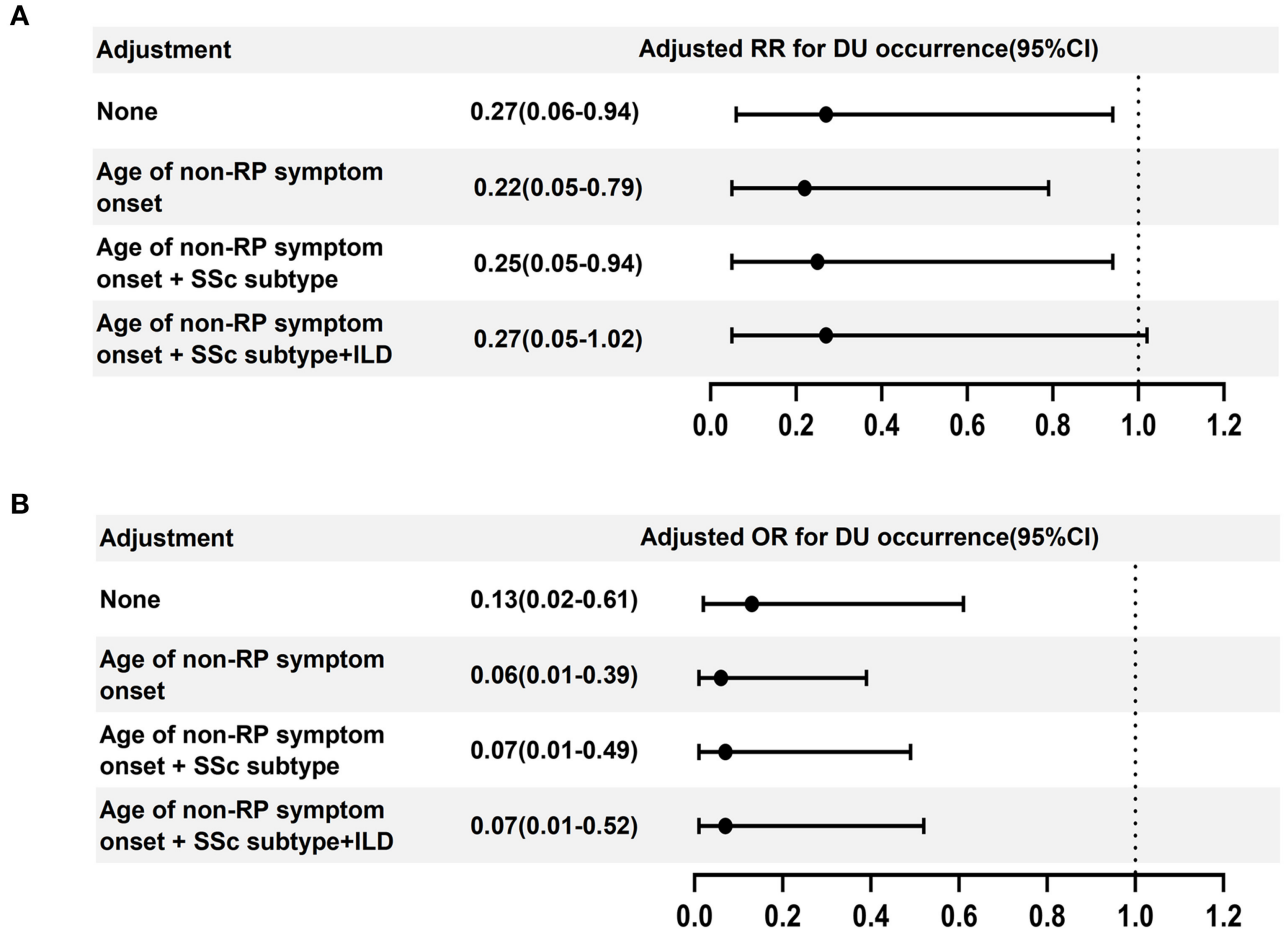
Forest plot displaying the association of the outcome (new occurrence of ischemic DUs) with IGU treatment. **(A)** aRR and 95% CI and **(B)** aOR and 95% CI of IGU treatment associated with DU occurrence. ILD, interstitial lung disease; CI, confidence intervals; DU, digital ulcer.

Both RR and OR for the IGU in preventing DU occurrence are presented in Figure 1. Overall, IGU usage was demonstrated to be effective in reducing the occurrence of DU. Adjusted by the age of SSc onset and skin subtype, exposure to IGU still appeared to be significantly associated with less occurrence of DU. With an additional adjustment for ILD, the OR was still significant, while the upper limit of 95% confidence interval of RR was slightly >1.

## Discussion

This is the first report of IGU as the treatment for SSc patients. As a disease-modifying drug for RA, IGU has been discovered to reduce inflammation via the nuclear factor (NF)-κ. IGU interferes with TNF-α-induced translocation of NF-κB and suppresses TNF-α-induced production of IL-6, IL-8, and monocyte chemoattractant protein 1 (MCP1) (32, 33). In addition, IGU selectively disturbs Act1–TRAF5 connections and TRAF5–Ikki interactions, interrupting IL-17 signaling (34). IGU inhibits macrophage migration inhibitory factor (MIF) tautomerase activity and prevents MIF-induced proinflammatory effects, therefore sparing steroids (35). COX-2 activity and transcription are both inhibited by IGU (36).

Besides anti-inflammation, recent studies shed some light on the anti-fibrotic effect of IGU. We have identified early growth response 1 (EGR1) as one of the most downregulated genes by IGU (20), which not only has an EGR1 signature in SSc but forms a positive feedback loop with TGF-β. Treatment with IGU effectively disrupts this loop. IGU also suppresses TGF-β signaling directly by partially inhibiting Smad2 phosphorylation (37). In addition, IGU has shown negative effects on matrix metalloproteinase-9, dampening the self-control of fibrosis in normal tissue (38).

In this preliminary observation, applied as a secondary treatment in most cases, IGU successfully prevented deterioration in 91.3% of cases. To our surprise, IGU demonstrated a positive effect on ischemic DU. In total, 72.7% (8/11) of IGU-treated patients had no new DU occurrence during follow-up with a median of 39 weeks (IQR: 10–96 weeks). In the clinical trials, the occurrence of DU was observed in 66.3% of patients with bosentan in 24 weeks (39) and 35.9–45.2% of subjects with macitentan in 16 weeks (30). DU is not only a major reason for patient bitterness (9) but a significant risk factor for cardiovascular complications of SSc (40) and a major component predictor of overall deterioration (23). Unfortunately, no immune suppressive treatment has been proven beneficial for ischemic DU.

Given these, we constructed a DU cohort to confirm the effect of IGU. We compared most of the known clinical risk factors for the new DUs, including the onset of the disease at a younger age (41–43), dcSSc (42, 44, 45), DU(s) ever (44, 45), elevated ESR (46), anti-TOPO (41, 47), presence of ILD (42), certain capillaroscopic phenotype (47, 48), and heavy smoking (>25 pack-years) (49), as well as some risk factors with conflicting results, such as sex (41, 46, 50), disease duration (42, 51), and presence of PH (42, 43). None of these factors was statistically different between patients with and without IGU therapy. This is a prospective cohort study that normally reports RR, but the sample size is small and the frequency of DU new occurrence is high, and it is more appropriate to describe diseases of high incidence with OR, therefore both RR and OR are presented. Adjusted by the age of disease onset and skin subtype, the RR and OR still significantly confirmed the protective effect of IGU on ischemic DU.

Regarding other clinical dimensions, we observed that all synovitis patients responded to IGU, as expected, with the effect of IGU in RA. As implied in a previous observation in RA-ILD, among the 13 SSc-ILD patients, six patients had improved FVC, four had stable lung function, and two had progression in ILD. Most patients (22/23) did not have mRSS progression, which fitted the natural history of SSc. Based on such a preliminary observation, it cannot be concluded whether IGU is beneficial for treating the lung and skin involvement of SSc. We did not analyze the GI involvement, although 18 of 23 patients had recorded symptoms and nine patients had records of GI symptomatic treatment (Supplementary Table). No escalation of GI treatment was observed. However, it was difficult to assess the IGU effect on GI involvement, as the symptoms were diverse and mild, as well as the symptomatic treatment was frequently adjusted during the follow-up.

No new side effects of IGU were observed in our SSc cohort. In total, five out of 23 patients had adverse events, including one mild anemia, one GI discomfort, one wheal response, and two liver injury (ALT less than three times the upper limit of normal), which all have been reported in previous RA studies (12, 52). Out of caution, we discontinued IGU in all five patients to optimize patient benefits.

Our study has strengths and limitations. Our study for the first time showed a hint of a DMARD beneficial for the treatment of SSc-DU. We do not have any clue on the mechanism of this phenomenon yet, with either our previous studies or other published literature on IGU cited above. This would be a very interesting question for the following research. Nevertheless, this is a preliminary single-center study with a small sample size, which might weaken the representativeness of the study population, such as the nominal high occurrence of new DU in the control group, compared with previous records (30, 39), and draws more caution to the result interpretation. Therefore, a larger, multi-center study is needed for further evidence. In addition, the descriptive part of the IGU efficacy on general SSc did not include a control group, although a statistically powerful control group for general SSc demands a huge sample size, given the high heterogeneity of the disease.

## Conclusion

For the first time, this study showed IGU treatment for SSc in the context of clinical practice. Although tested in a limited number of patients, it implies the possibility of IGU as an alternative treatment for SSc with acceptable tolerance and, unexpectedly, provides a hint of the first IS treatment protective of ischemic DU, which merits further investigation of IGU in SSc both clinically and scientifically.

## Data availability statement

The original contributions presented in the study are included in the article/Supplementary material, further inquiries can be directed to the corresponding authors.

## Ethics statement

The studies involving human participants were reviewed and approved by the Ethics Committee of Renji Hospital, Shanghai, China. The patients/participants provided their written informed consent to participate in this study.

## Author contributions

QRY and BL carried out the study and wrote the manuscript with support from JYW, HLY, and QQL. QRY and LJL initiated and designed the project. All authors discussed the results and contributed to the final manuscript, read, and approved the final manuscript.
