# Supplementary material for: Iguratimod as an alternative therapy for systemic sclerosis and prevention of the occurrence of ischemic digital ulcer
Source: Front Med (Lausanne). 2023 May 5;10:1113408. doi: 10.3389/fmed.2023.1113408 (PMC10196471; doi:10.3389/fmed.2023.1113408)
Supplement: Supplementary file 1 [file Data_Sheet_1.docx]

| Supplementary table. GI symptoms and symptomatic treatment for all patients | | |
| --- | --- | --- |
| Patient No. | GI symptom | Symptomatic treatment |
|  |  |  |
|  |  |  |
| 1 | Reflux | / |
| 2 | None | / |
| 3 | Diarrhea | Levofloxacin+rifaximin |
| 4 | Nausea, vomiting, and belching | Domperidone +mosapride→domperidone+azintamide |
| 5 | Reflux | / |
| 6 | Reflux | Rabeprazole |
| 7 | 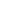None | / |
| 8 | Belching and stomachache | Azintamide |
| 9 | Nausea and vomiting | Domperidone |
| 10 | None | / |
| 11 | Bile reflux gastritis | Ursodeoxycholic acid |
| 12 | Occasional dysphagia | / |
| 13 | None | / |
| 14 | None | / |
| 15 | Diarrhea | / |
| 16 | Reflux | Rabeprazole |
| 17 | Nausea, vomiting, and reduced exhaust defecation | Linaclotide+parenteral nutrition |
| 18 | Nausea and belching | / |
| 19 | Nausea and belching | / |
| 20 | Reflux | Rabeprazole |
| 21 | Reflux | / |
| 22 | Nausea and vomiting | / |
| 23 | Belching | / |
